# Altered polyadenylation site usage in SERPINA1 3’UTR in response to cellular stress affects A1AT protein expression

**DOI:** 10.1038/s41598-025-07569-3

**Published:** 2025-07-02

**Authors:** F. N. U. Jiamutai, Abigail Hatfield, Austin Herbert, Debarati Majumdar, Vijay Shankar, Lela Lackey

**Affiliations:** Department of Genetics and Biochemistry, Center for Human Genetics, Clemson University, Clemson, USA

**Keywords:** Polyadenylation, RNA processing, Interleukin 6 (IL-6), Cirrhosis, SERPINA1, Alpha1 anti-trypsin (A1AT), RNA metabolism, Interleukins, Transcriptomics

## Abstract

Alternative polyadenylation results in different 3’ isoforms of messenger RNA (mRNA) transcripts. Alternative polyadenylation in the 3’ untranslated region (3’UTR) can alter RNA localization, stability and translational efficiency. The *SERPINA1* mRNA has two distinct 3’ UTR isoforms, both of which express the protease inhibitor α-1-antitrypsin (A1AT). A1AT is an acute phase protein that is expressed and secreted from liver hepatocytes and upregulated during inflammation. Low levels of A1AT in the lung contributes to chronic obstructive pulmonary disease, while misfolding of A1AT in the liver contributes to liver cirrhosis. We analyzed the dynamics of alternative polyadenylation during cellular stress by treating the liver cell line HepG2 with the cytokine interleukin 6 (IL-6), ethanol or peroxide. *SERPINA1* is transcriptionally upregulated after IL-6 treatment and has altered polyadenylation, resulting in an increase in long 3’UTR isoforms. We find that the long 3’UTR represses endogenous A1AT protein expression even with high levels of *SERPINA1* mRNA. *SERPINA1* expression and 3’ end processing are not affected by ethanol or peroxide. IL-6-induced changes in transcriptome-wide transcriptional regulation suggest changes to the endoplasmic reticulum and in secretory protein processing. Our data shows that inflammation influences polyA site choice for *SERPINA1* transcripts, resulting in reduced A1AT protein expression.

**Supplementary Information:**

The online version contains supplementary material available at 10.1038/s41598-025-07569-3.

## Introduction

To become mature transcripts the majority of human RNAs undergo 3’ end cleavage and polyadenylation (polyA)^1^. The polyA tail is important for RNA stability and translational efficiency^2^. Selection of the polyadenylation cleavage site determines the 3’ end of the transcript. In humans, many genes have multiple possible polyA sites and regulate their 3’ end RNA processing to produce alternative polyadenylation (APA) isoforms^3^. Alternative polyadenylation can result in messenger RNA (mRNA) transcripts with dramatically different 3’ UTR lengths and regulatory processes. Alternative polyadenylation can also occur in intronic or upstream coding exons, which can result in transcripts with premature stop codons or altered protein products. The mechanisms that control selection of alternative sites are not well understood. The expression of core polyadenylation proteins, accessory RNA binding proteins and RNA transcript structural accessibility can all influence site selection^4–6^. In some cases, processed RNA transcripts with longer 3’UTRs may be able to convert to shorter 3’UTRs, altering their regulatory properties, including stability, localization and translational potential^7,8^.

Most documented alternative polyadenylation is based on tissue specificity, but it is also a key part of cellular differentiation^4,9^. Control of alternative polyadenylation is cell-type specific, and pluripotent cells proliferating and differentiating into specific subtypes with less potency can undergo global 3’UTR lengthening or shortening^9^. For example, differentiation of cells into placenta trophoblasts is associated with global 3’ UTR shortening. Interestingly, trophoblast alternative polyadenylation genes are enriched in endoplasmic reticulum (ER) regulatory genes, consistent with a differentiated secretory role in the placenta^10^. In addition to differentiation, alternative polyadenylation is also associated with a dynamic response to cellular stressors^11,12^. Quiescent T cells converting to a proliferative state undergo alternative polyadenylation of genes associated with organelles and membrane trafficking^13^. Alternative polyadenylation, and specifically lengthening of 3’UTRs, is associated with cigarette smoking in white blood cells^14^. Alternative polyadenylation changes the 3’UTR sequencing of transcription and can impact protein expression. For example, polyA site choice can be RNA localization, such as in murine myoblasts where transcripts with longer 3’UTRs are more likely to be associated with the endoplasmic reticulum^15^.

Alternative polyadenylation in the *SERPINA1* precursor RNA was recently characterized and associated with tissue-specificity, with longer 3’UTR isoforms normally present in liver tissue^16^. The longer 3’UTR isoform of *SERPINA1* is also increased in lung tissues from patients with chronic obstructive pulmonary disease^16^. *SERPINA1* mRNA produces α-1-antitrypsin (A1AT) protein, which suppresses the immune response by inactivating neutrophil elastase^17^. A1AT is primarily translated in hepatocyte cells and secreted into the bloodstream where it can protect lung tissues from an overactive immune response^18^. *SERPINA1* is also expressed at low levels in lung tissues and white blood cells with different transcription start sites and 5’UTR splicing isoforms, resulting in complex post-transcriptional regulation^14,19–23^. Despite the importance of A1AT expression in liver tissue, a key gap in the field is how little we know about *SERPINA1* post-transcriptional regulation in this tissue.

*SERPINA1* is an acute phase protein, upregulated by inflammatory conditions, yet how these signals impact its post-transcriptional regulation in the liver is not known^18^. A1AT protein is translated from *SERPINA1* mRNA into the endoplasmic reticulum where it is post-translationally modified and secreted from liver cells^18,24,25^. Misfolding and ER stress of A1AT variants contribute to A1AT-associated disorders and can lead to chronic obstructive pulmonary disease and liver cirrhosis^26–31^. A1AT-related diseases are influenced by genetic variation and environmental conditions, like smoking and alcohol use^32–34^. Our central hypothesis is that inflammatory signals regulate *SERPINA1* alternative polyadenylation in liver cells. We tested whether inflammation and other stressors dynamically alter 3′ end processing of *SERPINA1*, potentially impacting A1AT protein output. We tested the impact of the inflammatory cytokine interleukin 6 (IL-6), ethanol and peroxide^35–37^. We used the HepG2 liver cell line as a model system to study both *SERPINA1* and transcriptome-wide RNA expression and alternative polyadenylation during cellular stress. We found that IL-6 exposure altered the regulation of secreted genes and endoplasmic reticulum-associated genes. IL-6 exposure specifically affected *SERPINA1*, causing an increase in the long 3’UTR isoforms. We confirmed that the long 3’UTR isoform of *SERPINA1* is less capable of producing A1AT protein. Neither ethanol or peroxide exposure affect *SERPINA1* expression or 3’ end processing, suggesting that not all cellular stressors act through the same regulatory mechanisms. Overall, our results show that inflammation alters transcriptional and post-transcriptional processes in liver cells, including regulation of *SERPINA1* polyadenylation, with implications for A1AT protein expression.

## Results

### Inflammation induced by IL-6 alters expression and 3’ end RNA processing of *SERPINA1*

**Fig. 1:**
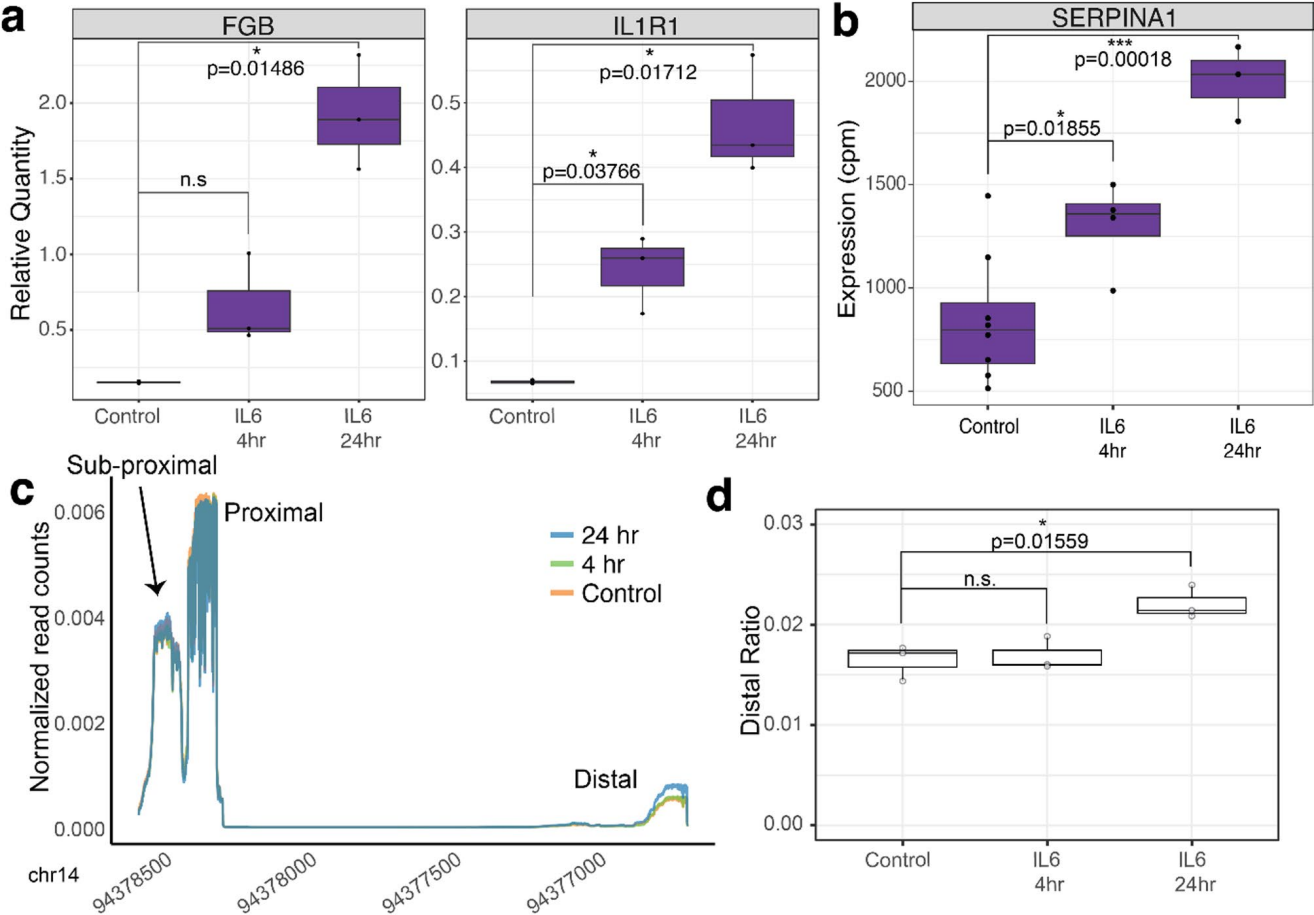
IL-6 induced inflammation changes *SERPINA1* RNA expression and processing in HepG2 liver cells. (**a**) IL-6 treatment at both 4 and 24 hours results in increased levels of *FGB* (24 hr p = 0.01486) and *IL1R1* (4 hr p = 0.03766, 24 hr p = 0.01712) mRNAs as measured by qRT-PCR normalized to GAPDH and t-test (**b**) and increased expression of *SERPINA1* mRNA as measured by RNA-seq (cpm) (4 hr p = 0.01855, 24 hr p = 0.00018, t-test). RNA sequencing data was analyzed with edgeR. (**c**) The majority of *SERPINA1* polyadenylation occurs at the proximal peak with additional APA at a distal site as measured by 3’ end specific sequencing. IL-6 treatment at 24 h results in an increase in isoforms using the distal site. Reads are normalized to total reads within the final 3’ exon. (**d**) Distal *SERPINA1* reads are significantly increased with 24 h of IL-6 treatment (*p* = 0.01559, t-test). Reads were quantified from 3’ end specific sequencing data.

Individuals with chronic obstructive pulmonary disease have an increase in long 3’UTR isoforms of *SERPINA1* in lung tissue, potentially due to chronic inflammatory conditions^16^. However, *SERPINA1* mRNA and the A1AT protein product are primarily expressed in the liver^18^. We analyzed the 3’UTR of *SERPINA1* from published primary hepatocytes treated with IL-6. The available data is not 3’end specific sequencing, thus it does not accurately quantify polyadenylation sites^38^. Analysis of the 3’UTR of *SERPINA1* shows a non-significant trend toward longer 3’UTR alternative polyadenylation isoforms (Supplemental Fig. 1b and c). The HepG2 liver cell line expresses *SERPINA1* mRNA and has elevated levels of *SERPINA1* mRNA in response to IL-6 at a lower level than seen in data from published primary hepatocytes treated with IL-6^38^ (Fig. 1b, Supplemental Fig. 1a). The expression and up-regulation of *SERPINA1* in HepG2 cells suggest that this cell line is a consistent model for understanding *SERPINA1* expression and processing in human liver cells. Thus, to test the impact of inflammation on 3’ end processing we treated HepG2 liver cells with the cytokine IL-6. We verified IL-6 induced inflammatory conditions using qRT-PCR for *FGB* and *IL1R1*, which are upregulated in cells exposed to IL-6^38^. As expected, we saw a significant increase in expression for both *FGB* and *IL1R1* after normalization to *GAPDH* at 24 h of IL-6 treatment (Fig. 1a, FGB *p* = 0.0148, IL1R1 *p* = 0.0171, t-test). To look specifically at polyadenylation site choice, we performed 3’ end specific Quant-Seq sequencing. QuantSeq has been validated with total RNA sequencing for both differential gene expression analysis and 3’ end identification^39^. In addition, we found that *FGB* and *IL1R1* were significantly upregulated in the RNA-seq results after IL-6 treatment, reflecting our qRT-PCR results (Supplementary Fig. 1d and e).

Next, we analyzed expression and processing of *SERPINA1* mRNA. We saw significant upregulation of *SERPINA1* at 4 and 24 hours post IL-6 treatment in HepG2 cells as analyzed by edgeR quantification of RNA-seq reads (Figs. 1b and 4hr p = 0.01855, 24hr p = 0.00018, t-test). *SERPINA1* is annotated with one polyadenylation site in NCBI RefSeq, corresponding to the long 3’UTR isoform (NM_000295.5)^40^. QuantSeq 3’ end specific sequencing shows evidence for both the proximal and distal polyadenylation sites in our HepG2 cell lines, resulting in short and long 3’UTR isoforms respectively (Fig. 1c). Both the proximal and distal polyA site isoforms are annotated in Ensembl (ENST00000636712.1 and ENST00000393087.9)^41^ and have been found in lung and liver tissues^16^. We identified a third peak (sub-proximal) at the very end or within the coding portion of the terminal exon that also corresponds to an annotated transcript in Ensembl (ENST00000449399.7), suggesting that *SERPINA1* may have three polyadenylation sites (Fig. 1c). We did not find evidence for additional coding or intronic alternative polyadenylation in the HepG2 transcriptome. Treatment of HepG2 cells with IL-6 causes a significant increase in the amount of long *SERPINA1* 3’UTR isoforms 24 h after IL-6 treatment as analyzed by quantifying reads at proximal and distal polyA sites and normalizing these reads to the number of reads in the final 3’ exon of *SERPINA1* (Fig. 1c, d, *p* = 0.01559, t-test). Processing of *SERPINA1* 3’UTRs was not significantly affected 4 h post-IL6 exposure.

**Fig. 2:**
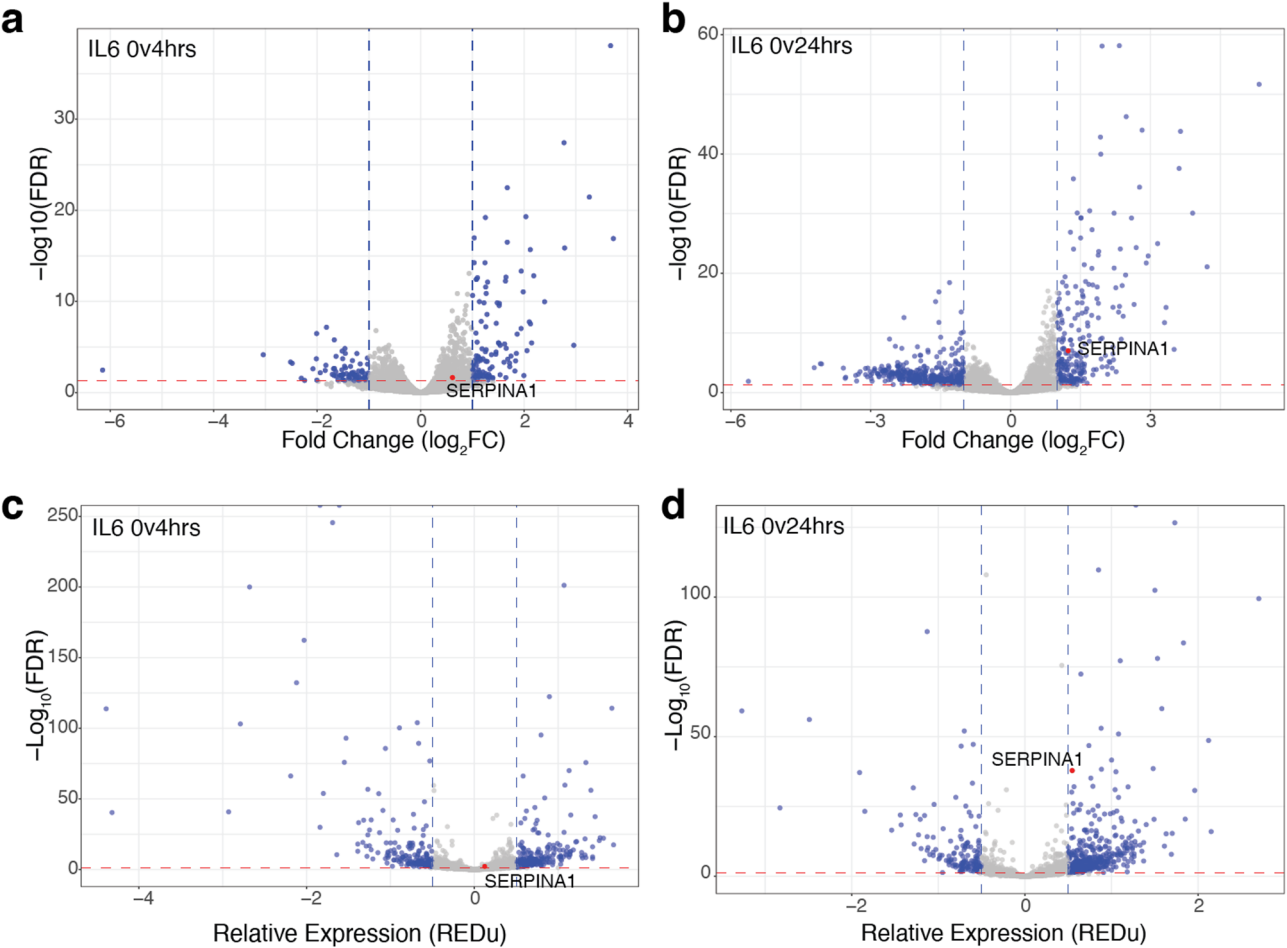
IL-6 induces transcriptome-wide reprogramming, including changes to 3’ RNA processing. Differential gene expression analysis after (**a**) 4-hours of IL-6 treatment and (**b**) 24-hours of IL-6 treatment showing transcriptome-wide changes as calculated by edgeR with FDR < 0.05, log_2_CPM > 6 and log_2_FC > 1. Alternative polyadenylation analysis with MAAPER after (**c**) 4-hours of IL-6 treatment and (**d**) 24-hours of IL-6 treatment showing transcriptome-wide changes with FDR < 0.05 and polyA score REDu +/-0.5. *SERPINA1* is highlighted in red.

### IL-6 induced inflammation influences transcriptome-wide expression and 3’ end processing

We identified 827 genes that are altered post-IL-6 exposure (edgeR, FDR < 0.05 and log_2_FC ±1) (Fig. 2a, b). In our transcriptome-wide analysis, *SERPINA1* mRNA is significantly upregulated after 24 h of IL-6 treatment (Fig. 2b, red, FDR = 9.82 × 10^− 8^). Approximately half of genes affected by IL-6 treatment at 4 h remain affected at 24 h (Supplementary Fig. 2a). Overall, more genes are downregulated after IL-6 treatment, especially 24 h post-IL-6 exposure, however, the median of expression changes is similar in magnitude at both 4- and 24-hour treatments (Supplementary Fig. 2b).

We used the program MAAPER to quantify alternative polyadenylation transcriptome-wide^42^. We found 806 genes with significant changes in polyA site choice at 4 and/or 24 h post-IL-6 treatment (FDR < 0.05 and REDu polyadenylation score +/- 0.5) (Fig. 2c, d). MAAPER identified *SERPINA1* as differentially polyadenylated after 24 h of IL-6 exposure (Fig. 2d, adj p-val = 1.4 × 10^− 38^). Approximately half of genes that undergo alternative polyadenylation at 4 h post-IL-6 treatment remain affected after 24 h (Supplementary Fig. 2c). RNAs become shorter and longer with similar magnitude (Supplementary Fig. 2d), however, more genes have longer 3’UTRs after IL-6 exposure. Both polyAdb- and polyAsite-derived polyA site annotations produce similar results for top genes with differential alternative polyadenylation^43,44^ (Supplemental Fig. 2e). There is no correlation between RNA levels and alternative polyadenylation in our analysis (Supplemental Fig. 2f). Only a small fraction of genes that are differentially expressed are also alternatively polyadenylated (Supplementary Fig. 2g).

**Fig. 3:**
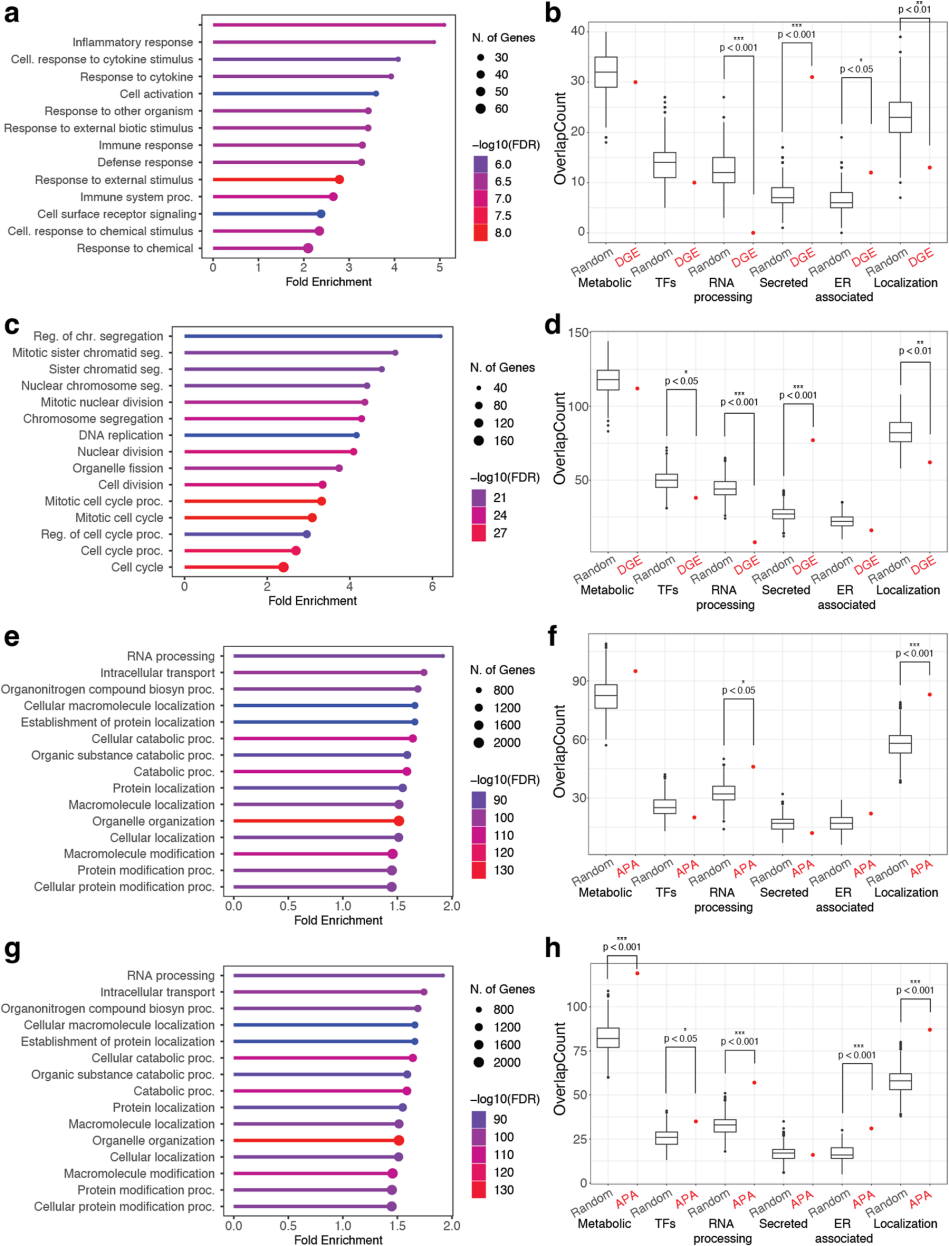
Endoplasmic reticulum genes are differentially expressed and alternatively polyadenylated during the inflammatory response. (**a**) Response to inflammation and the environment were top biological processes 4-hours post-IL-6 exposure (ShinyGO) while (**b**) secreted genes were overrepresented in gene sets (bootstrapping from all expressed genes, permutation analysis, reported as significance level). (**c**) 24-hours post-IL-6 exposure, cell cycle-associated biological processes were enriched (ShinyGo) and (**d**) secreted genes continued to be overrepresented by boot-strapping and permutation test calculations. APA analysis (**e**) 4-hours post-IL-6 exposure gene ontology (ShinyGo) and (**f**) gene-set overrepresentation identified RNA processing and protein localization-associated genes by bootstrapping and permutation test. Both RNA processing and protein localization genes were also identified by (**g**) ontology through ShinyGo and (**h**) enrichment analyses 24-hours post-IL6 exposure by boot-strapping and permutation test calculations. Significance levels *p* < 0.05*, *p* < 0.01**, *p* < 0.001***.

We found a variety of environmental responses, including the inflammatory and cytokine responses, enriched 4 h post-IL-6 treatment using gene ontology analysis for biological processes (Fig. 3a, ShinyGo). Gene list enrichment identified secreted genes and genes associated with protein localization as significantly more likely to be differentially expressed than expected (Human protein atlas - predicted secreted proteins and GO:0008104)^45,46^ (Fig. 3b, *p* < 0.001, permutation analysis). Metabolic-associated genes were not affected by IL-6 exposure (Human protein atlas - metabolic proteins) (Fig. 3b). The top gene ontology pathways shifted at 24 h post-IL-6 treatment to include cell division and cell cycle pathways (Fig. 3c, ShinyGO), but secreted genes and protein localization genes remained significantly more likely to be differentially expressed at 24 h post-IL-6 exposure (Fig. 3d, *p* < 0.001, permutation analysis). RNA processing genes were significantly depleted from differentially expressed genes at both 4- and 24-hours post-IL-6 treatment (GO:0006396)^46^ (Fig. 3b, d, *p* < 0.001, permutation analysis), suggesting that RNA processing genes are less likely to be differentially expressed than other genes. Significance was calculated for gene list enrichment against random gene sets using permutation analysis.

**Fig. 4:**
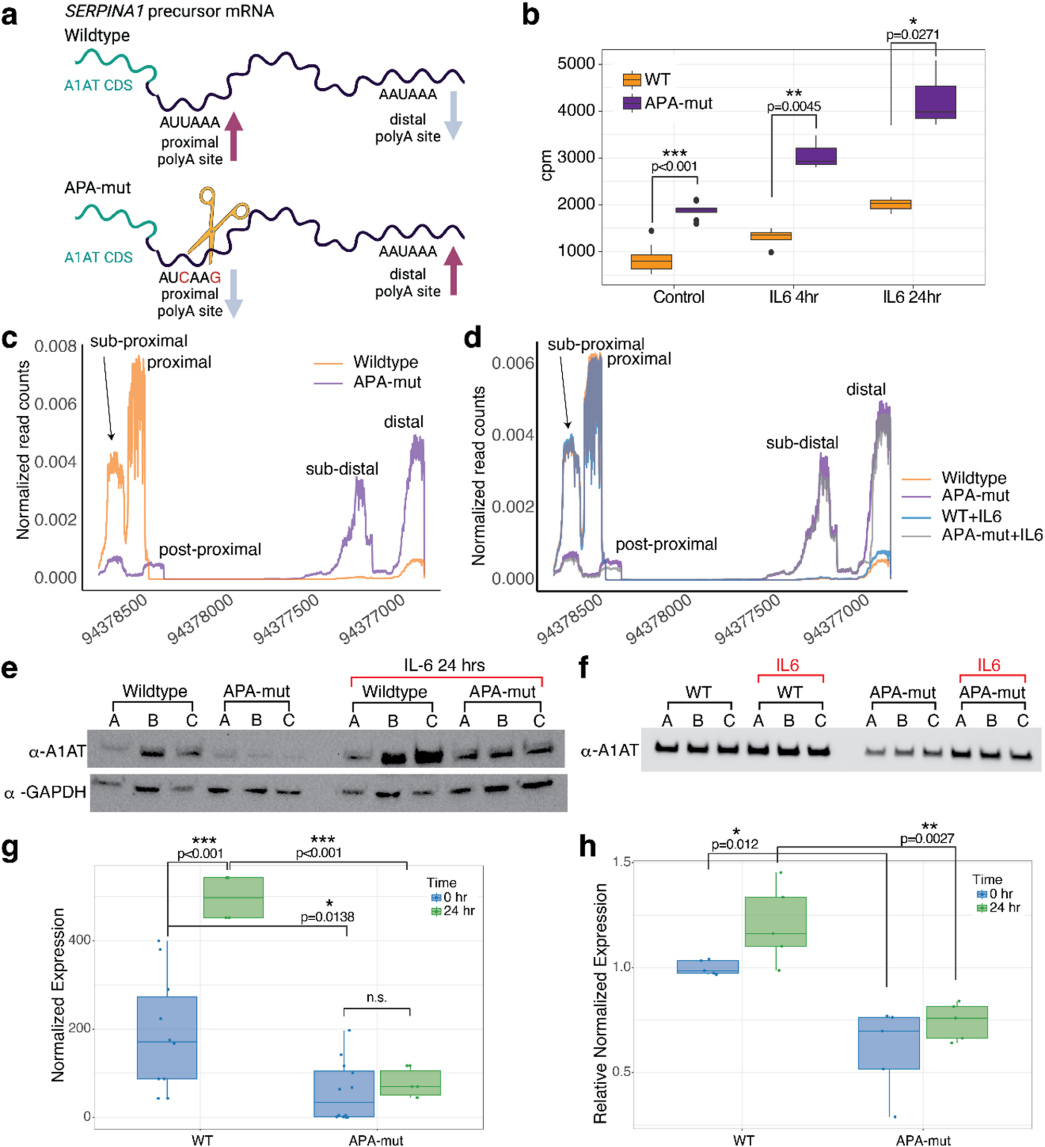
The long 3’UTR of *SERPINA1* represses A1AT protein expression. (**a**) APA-mut HepG2 cells were created by CRISPR mutation of the proximal polyA site from AAUAAA to AUCAG (Synthego). Figure created in Biorender. (**b**) APA-mut cells express significantly more *SERPINA1* mRNA than wild-type and increase expression upon IL-6 treatment as calculated from RNA-seq data with edgeR followed by t-test analysis for significance. (**c**) Mutation of the proximal polyA site in the APA-mut cell line (purple) increases the use of distal polyA sites. Reads are normalized to the total number of reads in the final 3’ exon. (**d**) Treatment of wildtype HepG2 cells results in an increase in distal polyA usage after 24-hours, but does not affect the ratio of distal polyA site use in APA-mut cells. (**e**) A1AT protein expression is decreased in the APA-mut cell line, as measured by western blot with A1AT antibody to wild-type and APA-mut cell lysate in triplicate. Expression of GAPDH was used as a normalization control. The same procedure was used to show that A1AT protein expression is upregulated after IL-6 treatment, but upregulation remains impaired in the APA-mut cell line. (**f**) Secreted A1AT protein is decreased in the APA-mut cell line with and without IL-6 treatment as measured by western blot with A1AT antibody. (**g**) Quantification of A1AT from cellular lysate using GAPDH protein as a normalization control. (**h**) Quantification of A1AT secreted into the cellular media using Ponceau staining as a normalization control. T-tests were used to calculate significance.

When we analyzed alternative polyadenylation data using gene ontology, we found enrichment for RNA processing at both 4- and 24-hours post-IL-6 treatment (Fig. 3e, g, ShinyGo). This enrichment was consistent in gene list analysis where RNA processing was more likely to be differentially polyadenylated compared to random (Fig. 3f–h and 4hr p < 0.05, 24hr p < 0.001, permutation analysis). These results suggest that RNA processing genes are affected by the inflammatory response at the post-transcriptional level but not at the transcriptional level. Genes associated with protein localization were more likely to be differentially expressed at both 4- and 24-hours post-IL6-exposure (Fig. 3f,h, p < 0.001, permutation analysis). After 24 hours of IL-6 exposure genes associated with the endoplasmic reticulum were significantly more likely to be alternatively polyadenylated (Human protein atlas – endoplasmic reticulum)^45^ (Fig. 3h, p < 0.001, permutation analysis). Both protein localization and endoplasmic reticulum-associated genes are likely affected by IL-6 through both transcriptional and post-transcriptional regulation, including 3’ processing. Inflammation-induced alternative polyadenylation of endoplasmic reticulum-associated genes is only evident after 24 h of exposure, suggesting a delayed response (Fig. 3h). Significance was calculated for gene list enrichment against random gene sets using permutation analysis. Previous studies have associated alternative polyadenylation with regulation of secreted proteins, such as within B cells and placental cells^10,15^. Our results indicate that liver cells may alter their secretory network during inflammation by controlling RNA levels and regulating RNA 3’ end processing.

### 3’ end RNA processing regulation in *SERPINA1* precursor mRNA affects A1AT protein expression

*SERPINA1* produces A1AT, which is secreted from liver hepatocytes. Previous research showed that the long 3’UTR isoform of *SERPINA1* was associated with repression of protein synthesis using nanoluciferase reporter assays^16^. We developed a *SERPINA1* CRISPR mutant cell line to determine whether 3’ end processing influences A1AT protein expression from the endogenous *SERPINA1* locus expressed in HepG2 liver cells (APA-mut line) (Fig. 4a). Treatment of wild-type and APA-mut HepG2 cells with IL-6 results in similar overall gene expression changes (Supplemental Fig. 3a-c). However, *SERPINA1* mRNA has higher expression in the APA-mut cell line than in wildtype HepG2s both before and after IL-6 treatment (t-test, Fig. 4b, Supplemental Fig. 3a-c, red points). The higher expression level of *SERPINA1* in APA-mut lines likely corresponds to a feedback loop that controls A1AT levels and has previously been shown to down-regulate gene expression in patients treated with exogenous A1AT^47^. APA-mut lines dramatically shift from predominately proximal polyadenylation of *SERPINA1* to predominantly distal polyA sites (Fig. 4c). As the sub-proximal polyadenylation site also decreases in expression, this polyadenylation may use the same polyA motif as the proximal site to guide alternative cleavage (Fig. 4c). Although the main proximal *SERPINA1* site is mutated and unable to be used for processing, we see rarely used post-proximal and sub-distal alternative polyadenylation sites that generate novel shorter 3’UTRs (Fig. 4c). Although *SERPINA1* mRNA is expressed at overall higher levels in APA-mut cells, IL-6 treatment induces a similar up-regulation of *SERPINA1* mRNA in both lines (Fig. 4b, Supplemental Fig. 3d and e, red points). However, IL-6 treatment of APA-mut cell lines does not increase the distal polyA site usage as does in wild-type HepG2 cells (Fig. 4d). Consistent with this, MAAPER transcriptome-wide analysis does not identify *SERPINA1* as alternatively polyadenylated after IL-6 treatment. The loss of IL-6 mediated 3’ processing when the proximal site is mutated implies that the increase in distal polyadenylation is controlled by regulation of the proximal polyA site. While differential gene expression is consistent between wild-type and APA-mut lines at 4- and 24-hours post-IL-6 exposure, alternative polyadenylation is not highly correlated 4 h post-IL-6 exposure (Supplemental Fig. 3f). Instead we only see high correlation 24 h post-IL-6 exposure, suggesting that alternative polyadenylation may be a longer-term regulation for IL-6 mediated inflammation in HepG2 cells (Supplemental Fig. 3g).

Reporter assays have shown that long 3’UTR isoforms of *SERPINA1* repress nanoluciferase protein expression^16^. Therefore, we tested A1AT protein production in the wildtype and APA-mut cell lines. Despite the higher levels of *SERPINA1* mRNA in APA-mut cells, A1AT protein levels were much lower in mutant cells, which primarily produce the long 3’UTR isoform of *SERPINA1* (Fig. 4e, *p* = 0.0138, Supplemental Fig. 4, t-test). A1AT protein expression is significantly upregulated after 24 h of IL-6 exposure (Fig. 4e, *p* = 4.3 × 10^− 5^, Supplemental Fig. 5). A1AT protein in the mutant line is not significantly upregulated after IL-6 treatment and remains at a much lower level than in wildtype HepG2 cells (Fig. 4e,g). Quantification of protein and RNA levels shows that 24 h of IL-6 exposure induces *SERPINA1* mRNA upregulation by 1.24-fold, while A1AT protein increases by 2.62-fold. In contrast, in APA-mut cells, while IL-6 exposure increases *SERPINA1* mRNA by a similar amount (1.18-fold), A1AT protein levels only increase by 1.32-fold (Fig. 4g). Since A1AT is secreted from hepatocytes, we tested the levels of A1AT protein in the media. We found significantly more A1AT secreted from wildtype HepG2 cells than from APA-mut HepG2 cells (Fig. 4f,h, *p* = 0.0027, t-test). IL-6 exposure showed a minor but non-significant increase for both wildtype and mutant cells (Fig. 4f, h), indicating that changes in *SERPINA1* mRNA levels and intracellular A1AT expression may not directly correlate with secreted A1AT. The inability of the APA-mut cell line to produce A1AT, especially in the presence of IL-6, directly links alternative polyadenylation to A1AT protein expression, emphasizing that increased long 3’UTR isoforms following IL-6 treatment reduce the capacity for A1AT protein synthesis.

### SERPINA1 is not affected by cellular stress induced by ethanol and peroxide exposure

**Fig. 5:**
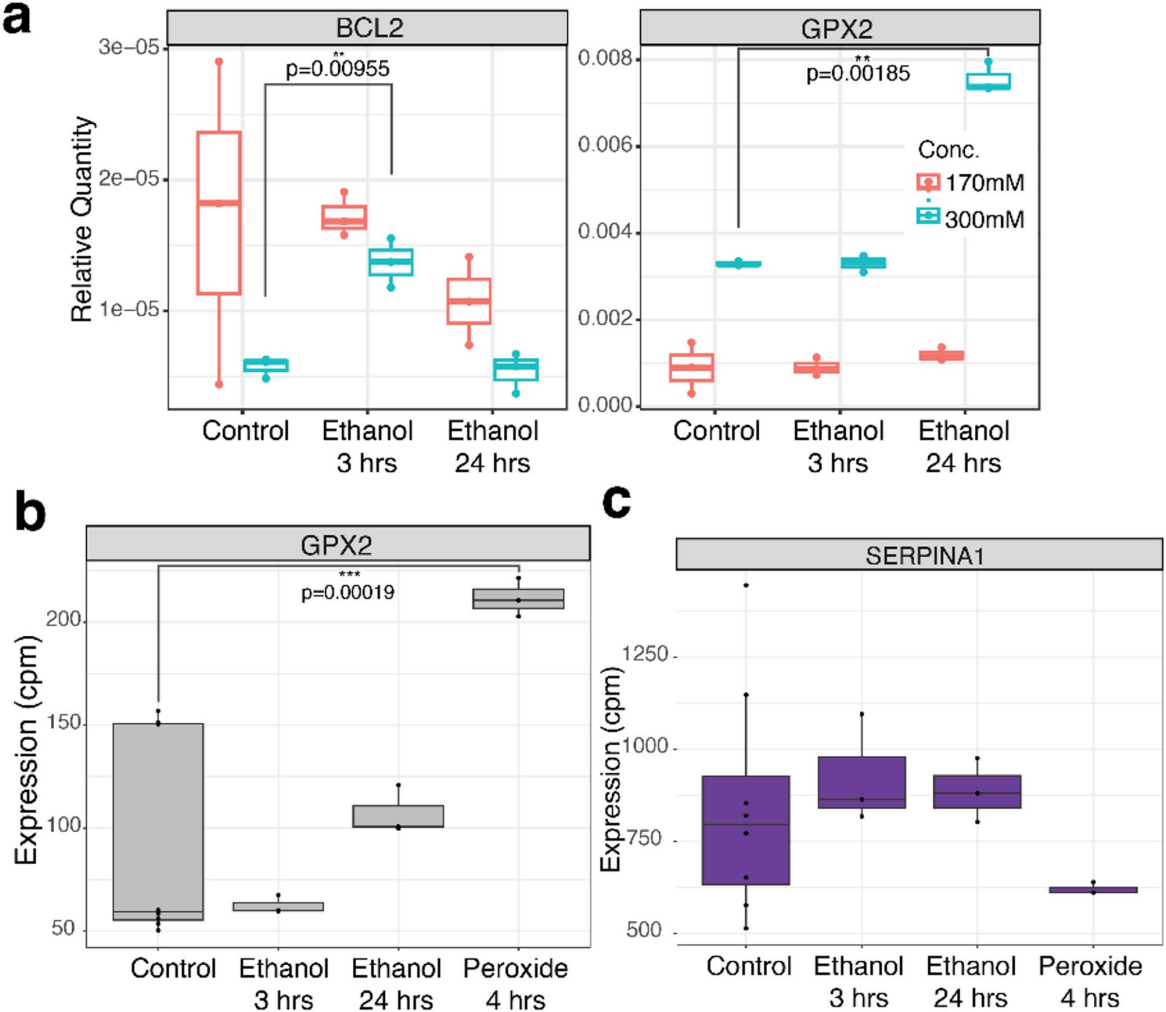
Cellular response to ethanol and peroxide does not alter *SERPINA1* mRNA expression. (**a**) *BCL2* and *GPX2* have moderate expression changes with ethanol treatment as quantified by qRT-PCR normalized to GAPDH. (**b**) RNA-seq data follows the same non-significant trend for *GPX2* expression with 170mM ethanol treatment, but shows upregulation of *GPX2* with peroxide exposure. (**c**) RNA-seq data of *SERPINA1* mRNA expression shows similar levels of mRNA at both 3- and 24-hours post-ethanol exposure and with peroxide treatment.

Liver stress can be caused by ethanol exposure^48,49^. Ethanol induces reactive oxygen species (ROS)^37^. To test the impact of acute ethanol exposure on 3’ end processing we treated cells with 170 mM or 300 mM ethanol for 3–24 h. We used qRT-PCR to analyze the effect of ethanol treatment on *BCL2* and *GPX2* mRNAs. When we treated cells with 300 mM ethanol, we saw a significant response to *BCL2* after 4 h of ethanol treatment and to *GPX2* at 24 h of ethanol exposure (t-test, Fig. 5a). This aligns with published literature on the *BCL2* and *GPX2* response to ROS^50,51^. We did not find a significant response to ethanol treatment with 170 mM ethanol, although the expression trend is similar between the two conditions. While 170 mM ethanol avoided ethanol-induced toxicity this level of treatment and acute time-scale may not have been enough to induce a strong cellular response. Additional studies are needed to fully understand the impact of ethanol on *SERPINA1* and the transcriptome. RNA-seq data for *GPX2* are consistent with qRT-PCR data, with a non-significant trend in increased expression with ethanol treatment (t-test, Fig. 5b). In addition to ethanol treatment, we also treated cells with peroxide directly to analyze the ROS pathway separate from other effects caused by ethanol exposure. *GPX2* is significantly upregulated with peroxide treatment, as expected^50^. *SERPINA1* expression is not significantly affected by ethanol or peroxide treatment (Fig. 5c).

**Fig. 6:**
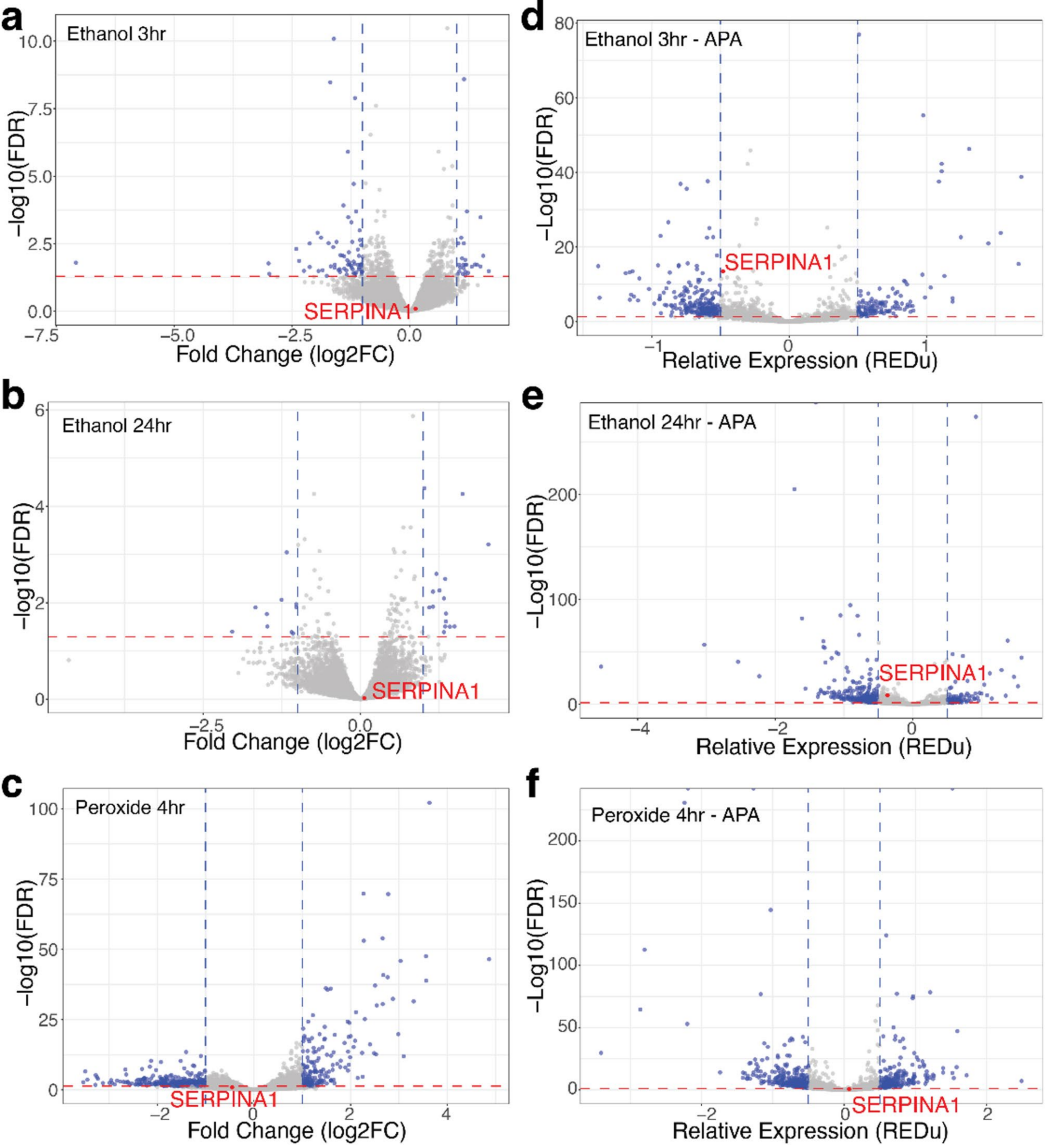
Ethanol and peroxide exposure do not affect *SERPINA1* expression or 3’ end processing. Ethanol treatment at 170mM for (**a**) 3-hours or (**b**) 24-hours alters gene expression as measured by RNA levels. *SERPINA1* is unaffected (red). (**c**) Peroxide exposure over four hours affects gene expression transcriptome-wide, but does not significantly alter *SERPINA1* mRNA levels (red). (**d**) Transcriptome-wide alternative polyadenylation is altered following (**d**) 3-hours or (**e**) 24-hours of 170mM ethanol treatment or (**f**) 4 h of peroxide exposure. Significant differential expression was identified using edgeR with FDR < 0.05, log_2_CPM > 6 and log_2_FC > 1. *SERPINA1* mRNA alternative polyadenylation is not significantly impacted with ethanol or peroxide treatments (red). Significant alterations in polyadenylation were identified with MAAPER with FDR < 0.05 and polyA score REDu +/-0.5.

Ethanol caused mild perturbation to the transcriptome with 96 and 26 differentially expressed genes at 3 and 24 h respectively (FDR < 0.05 and log_2_FC +/-1) (Fig. 6a,b). Only a small number of genes are differentially expressed at both 3 and 24 h (Supplemental Fig. 7a), however, these genes are consistently up or down regulated (Supplemental Fig. 7b, log_2_FC +/-0.5). In contrast, our peroxide treatment was harsher and had a major impact on the transcriptome, with 572 genes differentially expressed after 4 h of treatment (FDR < 0.05 and log_2_FC +/-1) (Fig. 6c). Ethanol and peroxide treatment have minimal overlap in the number of differentially expressed genes (Supplemental Fig. 7c).

*SERPINA1* 3’ end processing is not altered by ethanol or peroxide exposure (Fig. 6d–f). However, both ethanol and peroxide impact RNA 3’ processing transcriptome-wide, with ethanol affecting alternative polyadenylation for 581 genes (Fig. 6d, e) and peroxide affecting alternative polyadenylation for 530 genes (Fig. 6f) (FDR < 0.05 and REDu ±0.5). Approximately a third of genes with differential polyadenylation 3 h post-ethanol exposure remain differentially polyadenylated at 24 h post-ethanol exposure (Supplemental Fig. 7d). Some genes that are alternatively polyadenylated by ethanol treatment are also alternatively polyadenylated with peroxide exposure (Supplemental Fig. 7e). Ethanol and peroxide have a minor impact on the endoplasmic reticulum and secreted genes although the response is not as consistent or strong as IL-6 exposure (Supplemental Fig. 8a–f).

## Discussion

Alternative polyadenylation has been linked to secretory pathways^10^. Liver hepatocytes have an important secretory role, producing high levels of secreted serum proteins^52^. In addition, inflammation induces upregulation of acute phase proteins like A1AT, which must be synthesized and secreted from the liver^52^. The connection between alternative polyadenylation and secretory pathways is consistent with our results, where we find that inflammation causes changes to the transcriptome at the transcriptional and post-transcriptional level that impact endoplasmic reticulum processes and secreted genes (Fig. 3, Supplemental Tables 2 and 3). We explored how genes in this pathway might impact the liver. The genes *P4HA1* and *Sect. 63* are part of the endoplasmic reticulum pathway defined by the Human Protein Atlas^45^ and are alternatively polyadenylated under inflammatory conditions (Supplemental Table 3). P4HA1 is involved in collagen biosynthesis and part of the prolyl-4-hydroxylase complex, which is a diagnostic biomarker in non-alcholic fatty acid liver disease and associated with hepatic fibrosis^53^. Similarly, we found alternative polyadenylation in *Sect. 63*, which necessary for co-translational protein translocation and associated with cholestatic liver disease when mutated^54^. Additionally, secretory-related genes like *TFPI*,* TGFBR2* and *SORL1* show polyadenylation changes under inflammatory conditions (Supplemental Table 2). TFPI (Tissue Factor Pathway Inhibitor) is a secreted protein that helps manage coagulation and inflammation. This gene has been shown to be differentially expressed in liver fibrosis^55^. TGFBR2 is a key factor in TGF-β signaling pathways which can influence liver regeneration, fibrosis, and hepatocellular carcinoma^56^. *SORL1* is a sorting receptor involved in endosomal trafficking and autophagy and can be altered in alpha-1-antitrypsin deficiency^57^. Together, these findings highlight the intersection of alternative polyadenylation, ER function, and secretory pathway genes in shaping the liver’s response to inflammation.

Since acute phase genes are also secreted, we analyzed these genes as well. *SERPINA1* mRNA levels and other known acute phase genes increase with IL-6 treatment (Supplemental Fig. 9a). We did not find most acute phase genes to have altered polyA site choice by MAAPER, however, when we directly analyzed 3’UTR reads in all acute phase genes we manually identified additional upregulated acute phase genes - ceruloplasmin (*CP)* and haptoglobin (*HP)* as alternatively polyadenylated (Supplemental Fig. 9b). While the polyA choice for most acute phase genes remains the same with IL-6 exposure (11/14 genes) (Supplemental Fig. 9c), our analysis does show that alternative polyadenylation regulates a substantial fraction of acute phase genes during inflammation (~ 20%). Additional work is needed to develop comprehensive 3’ end annotation sets for alternative polyadenylation analysis.

As human liver is a difficult system to study, we used the HepG2 cell line as a model system. We confirmed that HepG2 cells behave similarly to primary human hepatocytes with IL-6 exposure with upregulation of *SERPINA*^38^. In addition, we have previously confirmed the existence of *SERPINA1* long and short 3’UTR isoforms in primary liver and lung tissues^16^. Future studies may be able to more directly access human tissues and study alternative polyadenylation in an more complex or primary context. For example, treatment of HepG2 cells with chemical stressors cannot replicate the environment of the human liver where immune cells co-exist with hepatocytes and supporting liver cell types^58^. Additional work is also necessary to fully understand the impact of ethanol and reactive oxygen species on the liver transcription. While we did not find an impact of ethanol or peroxide treatment on *SERPINA1* expression or 3’ end processing, this may be due to our short-term, simplified treatment model. A better understanding of the impact of ethanol on 3’ end processing and *SERPINA1* regulation in hepatocytes may require 3D organoid systems, a chronic ethanol exposure time-course or co-exposure of ethanol with the pro-apoptotic cytokine TGF-ß^59^.

In *SERPINA1*, mutation of the proximal polyA site prevents IL-6 mediated 3’UTR regulation. This suggests that the proximal polyA site is the primary regulatory target and the increase in long 3’UTR isoforms results from repression of the proximal polyA site. The *SERPINA1* long 3’UTR isoform is less capable of producing A1AT protein. Our data suggest that while *SERPINA1* mRNAs are upregulated during the early inflammatory response, A1AT protein production is muted by non-productive long 3’UTR transcripts. This allows the cells to initiate high levels of RNA transcription but reduced translation of A1AT and potentially alleviate an overburdened secretory system. Recently it has been shown that long 3’UTR transcripts can be converted to short 3’UTR isoforms, suggesting that one interesting future avenue of research would be to understand whether *SERPINA1* long 3’UTRs can be converted to productive short 3’UTR isoforms^7,8^.

The mechanism underlying *SERPINA1* long 3’UTR isoform repression remains unknown, but several RNA binding proteins have been linked to A1AT repression, including QKI and NQO1^16,60^. In addition, the miR-320 family has been implicated in translational regulation of A1AT expression during inflammation^61^. Exogenous A1AT protein represses endogenous production of *SERPINA1*, suggesting the existence of a negative feedback loop and highlighting the importance of both transcriptional and post-transcriptional control of *SERPINA1* translation of A1AT^47^. Evidence of the feedback loop of A1AT expression in *SERPINA1* gene expression is supported by the increase in post-proximal alternative polyadenylation, which produces transcripts with intermediary 3’UTR lengths that may be less repressive. These isoforms of *SERPINA1* are not expressed in normal HepG2 cells and may be driven by the impaired ability of long 3’UTR *SERPINA1* isoforms to produce A1AT protein. Additionally, upregulation of A1AT protein after IL-6 surpasses the upregulation of the mRNA, implying that post-transcriptional regulation may also act on the shorter 3’UTR isoforms of SERPINA1 to control A1AT translation.

A1AT deficiency is linked to a rare variant that causes A1AT misfolding and endoplasmic reticulum stress in the liver^31^. Our work identifies how inflammation and other cellular stressors influence gene expression and 3’ end processing of *SERPINA1* and other genes. We find that inflammation changes transcriptome-wide RNA expression and 3’ end processing for genes associated with the endoplasmic reticulum and protein localization. Remodeling of these secretory pathways during inflammation may contribute to disease and provide therapeutic targets to alleviate specific pathology, such as the role of A1AT misfolding and accumulation in hepatocytes in liver cirrhosis. Future studies on mechanism are necessary to understand how inflammation causes alternative polyadenylation of endoplasmic reticulum and protein localization genes and the functional impact of these APA events.

## Methods

### Cell culture

APA-mut cell lines were created through Synthego by CRISPR mediated mutation of AUUAAA to AUCAAG on the minus strand of chr14:94,378,388 − 94,378,393 (hg38 genome). HepG2 wildtype cells were also provided from Synthego and regularly checked for mycoplasma contamination (PCR Mycoplasma Detection Kit, ABM). Cell lines were seeded in 10 cm^2^ plates and grown at 5% CO^2^ and 37 C. Cells were grown in Eagle’s Minimum Essential Media (EMEM) supplemented with 5% FBS (Thermo Scientific) and 0.5% Penicillin-Streptomycin (MilliporeSigma). For exposure treatments, cell lines were seeded into at 3.0 × 10^5 cells in 6-well plates. Cells were incubated for 24 h then serum-starved using 1 mL serum-free EMEM for another 24 h. After media starvation, the cells were treated in 1 mL serum-free media with 170mM ethanol diluted in PBS (EtOH, 3, and 24 h) (Fisher Scientific), 150 μm hydrogen peroxide diluted in PBS (H2O2, 4 h) (Fisher Scientific) or 2.88 nM (60ng/mL) interleukin-6 resuspended in 0.1% of BSA (IL-6 4, and 24 h) (Fisher Scientific).

### RNA extraction

Total RNA was extracted using Trizol (Thermo Fisher) according to manufacturer’s recommendations for adherent cell lines using Phaselock columns (Quantabio) prior to RNA purification with RNA clean up columns (NEB). Genomic DNA was removed using TURBO DNase (Fisher Scientific) according to the given protocol. RNA was quantified via NanoDrop (Thermo Scientific ND-8000) and quality checked by RNA Screentape on a Tapestation 4150 (Agilent).

### Quantitative reverse transcriptase polymerase chain reaction (qRT-PCR)

qRT-PCR was performed on a QuantStudio 3 (Thermo Scientific) system using iTaq Universal SYBR Green One-Step Kit (Biorad). 150 ng of total RNA was loaded, in biological triplicate, into a total reaction of 20 uL. qRT-PCR run parameters were as follow: 10 min reverse transcription at 50 C, 1 min polymerase activation and DNA denaturation at 95 C, followed by 40 cycles involving denaturation at 95 C for 15 s, annealing/extension/plate read at 60 C for 1 min, and a melt curve analysis (95 C, 15 s; 60 C, 1 min; 95 C, 1 s with 0.15 C /s ramp rate). The sequences for the primers used for genes *BCL2*,* GPX2*,* GAPDH*,* ACTB*,* FGB*, and *IL1R1* are provided (Supplemental Table 1). At least three biological replicates were quantified. The software qRAT (version 0.2.0)^62^ was used to normalize test genes to GAPDH and calculate relative quantity (2^-deltaCq). T-tests was used to determine statistical significance.

### RNA library preparation and sequencing

RNA samples were prepared using the QuantSeq 3’ mRNA REV Library Prep Kit V2 (Lexogen), which captures polyadenylated RNA. Quality assurance for loading samples for sequencing was performed using a Qubit dsDNA High Sensitivity Assay (Thermo Scientific) on a Qubit 4 fluorometer (Thermo Scientific), and a High Sensitivity D1000 Screentape (Agilent) on a Tapestation 4150 (Agilent). The Lexogen QuantSeq samples were sequenced on a NovaSeq 6000 (Illumina) using a 2 × 100 cycle cartridge. A custom sequence primer was used for Read 1 according to instructions from Lexogen and Illumina.

### Quantification of APA events

RNA sequencing paired-end reads were aligned to the Homo sapiens reference genome (GRCh38.p13) using STAR (v2.7.10a). At least three biological replicates were used for each condition (WT HepG2 *n* = 8, IL6 4 h *n* = 4, IL6 24 h *n* = 3, ethanol 3 h *n* = 3, ethanol 24 h *n* = 3, peroxide 4 h *n* = 3). The genome build excluded ALT, HLA, and decoy regions. The genome annotation was sourced from Gencode release v39 (GTF: gencode.v39.GRCh38.ERCC.genes.gtf). A STAR genome index was generated using the --runMode genomeGenerate command. STAR alignment used the following key parameters: maximum allowed mismatches per read length (--outFilterMismatchNoverLmax 0.6), minimum required alignment score per read length (--outFilterScoreMinOverLread 0.33), and intron length constraints (--alignIntronMin 20, --alignIntronMax 1,000,000, --alignMatesGapMax 1,000,000). Multimapping reads were restricted to a maximum of 200 loci (--outFilterMultimapNmax 200), and collapsed splice junctions were limited to 5,000,000 (--limitOutSJcollapsed 5,000,000).

Direct coverage of select regions were conducted through Samtools mpileup counts^63^. Each position’s normalized read count was calculated by dividing total read counts of region from the read counts of each individual position. To calculate the length normalized ratio of reads within distal and proximal sections of the 3’UTR we divided the read counts by the length of the region and then divided this length normalized value by the sum of the read ratios of all regions. For transcript-wide APA analysis, we aligned reads based on QuantSeq and MAAPER^42^ guidelines for the second read pair only using STAR to hg38 with Gencode v39 annotations. The core STAR command used included the paramters --outFilterType BySJout --outFilterMultimapNmax 200 --outFilterMatchNminOverLread 0.33 --outFilterScoreMinOverLread 0.33 --alignSJoverhangMin 8 --alignSJDBoverhangMin 1 --outFilterMismatchNmax 999 --outFilterMismatchNoverLmax 0.6 --alignIntronMin 20 --alignIntronMax 1,000,000 --alignMatesGapMax 1,000,000 --limitOutSJcollapsed 5,000,000 --quantMode TranscriptomeSAM --outSAMattributes NH HI NM MD --outSAMtype BAM Unsorted. We used the R package MAAPER^42^ with read length of 100 and num_pas_thre of 50 to analyze aligned reads. We modified the polyAdb annotations^43^ provided with MAAPER to include the APA sites originally unannotated in *SERPINA1.* In addition, we used MAAPER with annotations from the polyAsite database^44^. To adjust the significance for multiple testing we used Benjamini and Hochberg’s False Discovery Rate correction^64^. Unless otherwise stated, differential polyadenylation was determined as genes with FDR < 0.05 and polyA score REDu greater or less than 0.5. Differentially polyadenylated genes were used as input into shinyGO for biological pathway analysis^65^. Differentially polyadenylated genes were used to compare against randomly selected HepG2 expressed genes using bootstrapping analysis for enrichment in gene lists from amiGO^46^ and the Human Protein Atlas (proteinatlas.org)^45^. Permutation tests were used to determine statistical significance and reported as p-value < 0.05, 0.01 or 0.001.

### Differential gene expression analysis

For differential gene expression analysis we used paired end alignment in STAR as described above followed by an edgeR-based pipeline for TMM normalization and quantification (available on GitHub https://github.com/vshanka23/snakemake_rnaseq), and expression modeling (“RNA-seq with edgeR”, https://github.com/bmunn99/Clemson-Grad-Rscripts). The edgeR package was used to normalize library sizes and quantify expression levels for individual genes^66^. At least three biological replicates were used for each condition (WT HepG2 *n* = 8, IL6 4 h *n* = 4, IL6 24 h *n* = 3, ethanol 3 h *n* = 3, ethanol 24 h *n* = 3, peroxide 4 h *n* = 3). Differentially expressed genes passed the following filters unless otherwise stated: FDR < 0.05, log_2_CPM > 6 and log_2_FC > 1. Differentially expressed genes were used as input into shinyGO for biological pathway analysis^65^. Differentially expressed genes were used to compare against randomly selected HepG2 expressed genes using bootstrapping analysis for enrichment in gene lists from amiGO^46^ and the Human Protein Atlas (proteinatlas.org)^45^. Permutation tests were used to determine statistical significance and reported as p-value < 0.05, 0.01 or 0.001.

### Western blot analysis

HepG2 cells were harvested using TrypLE, spun down at 500 x g, then resuspended in 500uL NP-40 buffer, with the addition of HALT Protease Inhibitor (Thermo Scientific). Column concentrators were used for media concentration which was volume normalized (Pierce Concentrator PES 10 K MWCO). Cells were lysed for 20 min on ice. Lysis was reduced and denatured using 4X BOLT LDS Sample Buffer (Novex). Samples were vortexed then denatured at 70 °C for 10 min then run on Bolt 10% Bis-Tris Plus Wedgewell Gels (Invitrogen) in 1X MES SDS Running Buffer (Invitrogen). Semi dry transfer was performed using the iBlot2 Gel Transfer Device (Invitrogen) with Iblot 2 NC Mini Stack (Invitrogen). Membrane was stained with a Ponceau S solution (Sigma) and imaged for total protein load on a Chemidoc MP (Biorad). The Ponceau S was removed (0.1% NaOH in nuclease-free water) and the membrane blocked for one hour with (5% fat-free milk, 1X TBS-T buffer). Blots were incubated with A1AT antibody (ThermoFisher #MA5-14661, 1:1000) for two hours then washed with TBS-T. Membranes were incubated with anti-mouse IgG HRP linked secondary (Cell Signaling Technology #7076, 1:2000) for one hour. This procedure was repeated for GAPDH (Santa Cruz #SC-32233, 1:2000) with the same secondary. The blots were washed then incubated in Clarity Western ECL substrate (BioRad) for five minutes. The proteins of interest were imaged using a Chemidoc MP on the Chemiluminescent setting with appropriate exposure times. Protein band quantification was performed using Image Lab software (version 6.1) and signals were normalized to GAPDH or Ponceau S protein expression. At least three biological replicates were quantified and a t-test used to determine statistical significance.

## Electronic supplementary material

Below is the link to the electronic supplementary material.

Supplementary Material 1

Supplementary Material 2

Supplementary Material 3

## Data Availability

All RNA-seq data are submitted at the Gene Expression Omnibus (GEO) and are available at GSE277810. Additional datasets used for comparative analysis are publicly available from the GEO at GSE202045.
