# Supplementary material for: Altered polyadenylation site usage in SERPINA1 3’UTR in response to cellular stress affects A1AT protein expression
Source: Sci Rep. 2025 Jul 2;15:23510. doi: 10.1038/s41598-025-07569-3 (PMC12222872; doi:10.1038/s41598-025-07569-3)
Supplement: Supplementary file 1 — Supplementary Material 1 [file 41598_2025_7569_MOESM1_ESM.pdf]

Supplemental Figures

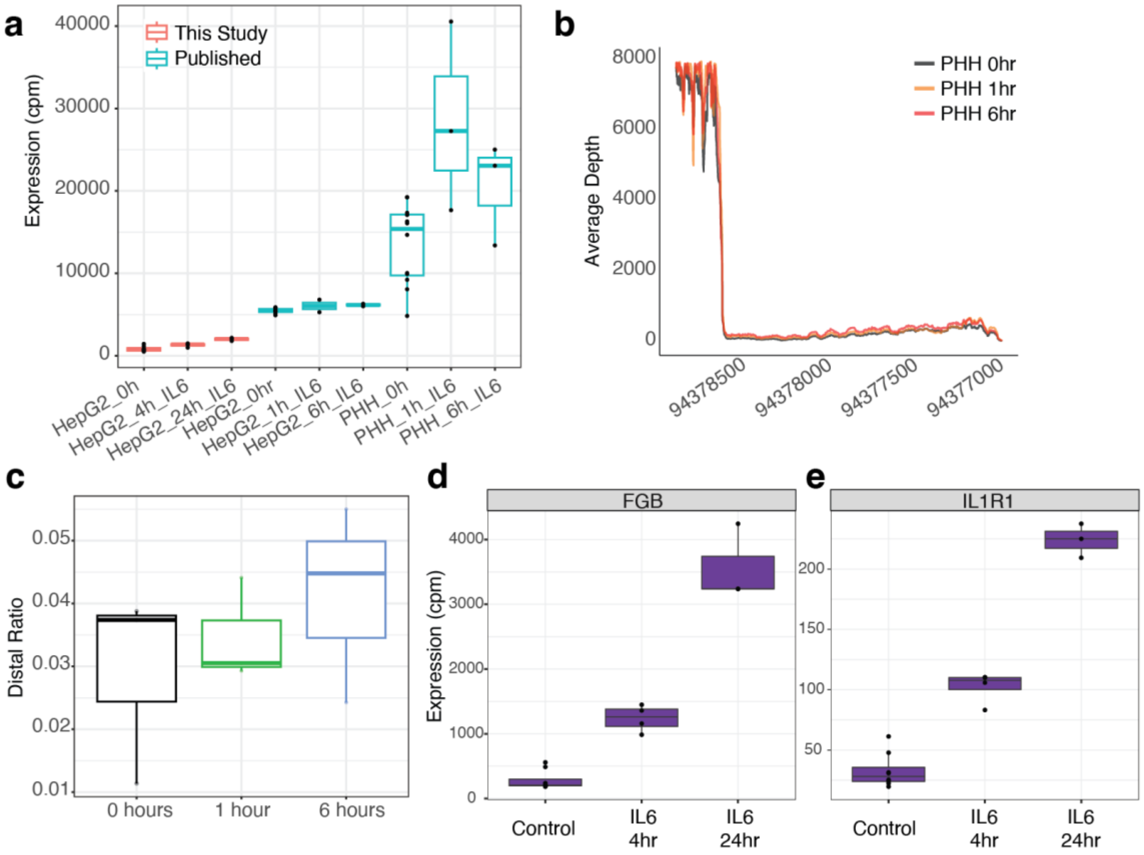

Supplemental Figure 1. Expression and processing in HepG2 and primary human hepatocytes of *SERPINA1*, *FGB* and *IL1R1*. (a) Increased *SERPINA1* expression in HepG2 cells before and after 4 hrs or 24 hrs of IL-6 exposure (this study), *SERPINA1* expression in HepG2 cells and primary human hepatocytes (PHH) before and after 1 and 6 hrs of IL-6 exposure (published, GSE202045). 3' end processing in *SERPINA1* in primary human hepatocytes (b) read depth from RNA-seq data and (c) quantification by proximal and distal regions does not identify clear differences before and after IL-6 treatment. Upregulation of (d) *FGB* and (e) *IL1R1* after IL-6 treatment of HepG2 in Quant-Seq data is consistent with qRT-PCR quantification.

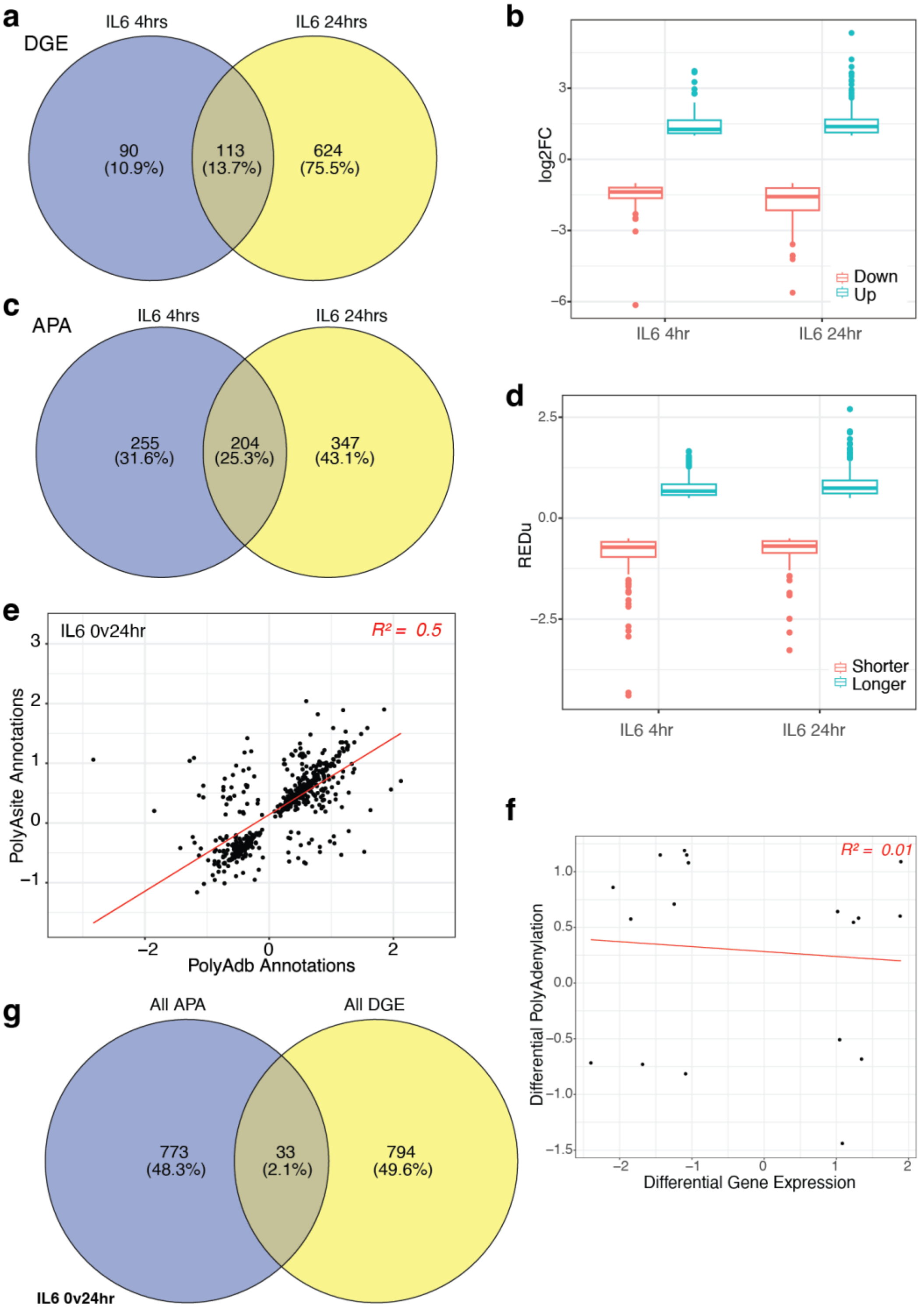

Supplemental Figure 2. Properties of genes that undergo IL-6 induced differential expression and alternative polyadenylation. (a) Overlap in genes identified as differentially expressed (DGE) at 4hr and 24hr post-IL-6 treatment and (b) the extent of up or down-regulation at each time point. (c) Overlap in APA genes at 4hr and 24hr post-IL-6 treatment and (d) the extent of 3’UTR shortening or lengthening. (e) Correlation between APA genes using either polyAdb or polyAsite annotations. (f) APA and gene expression are not correlated 24hrs post-IL-6 exposure. (g) Only a small number of genes that are significantly alternatively polyadenylated are also significantly differentially expressed.

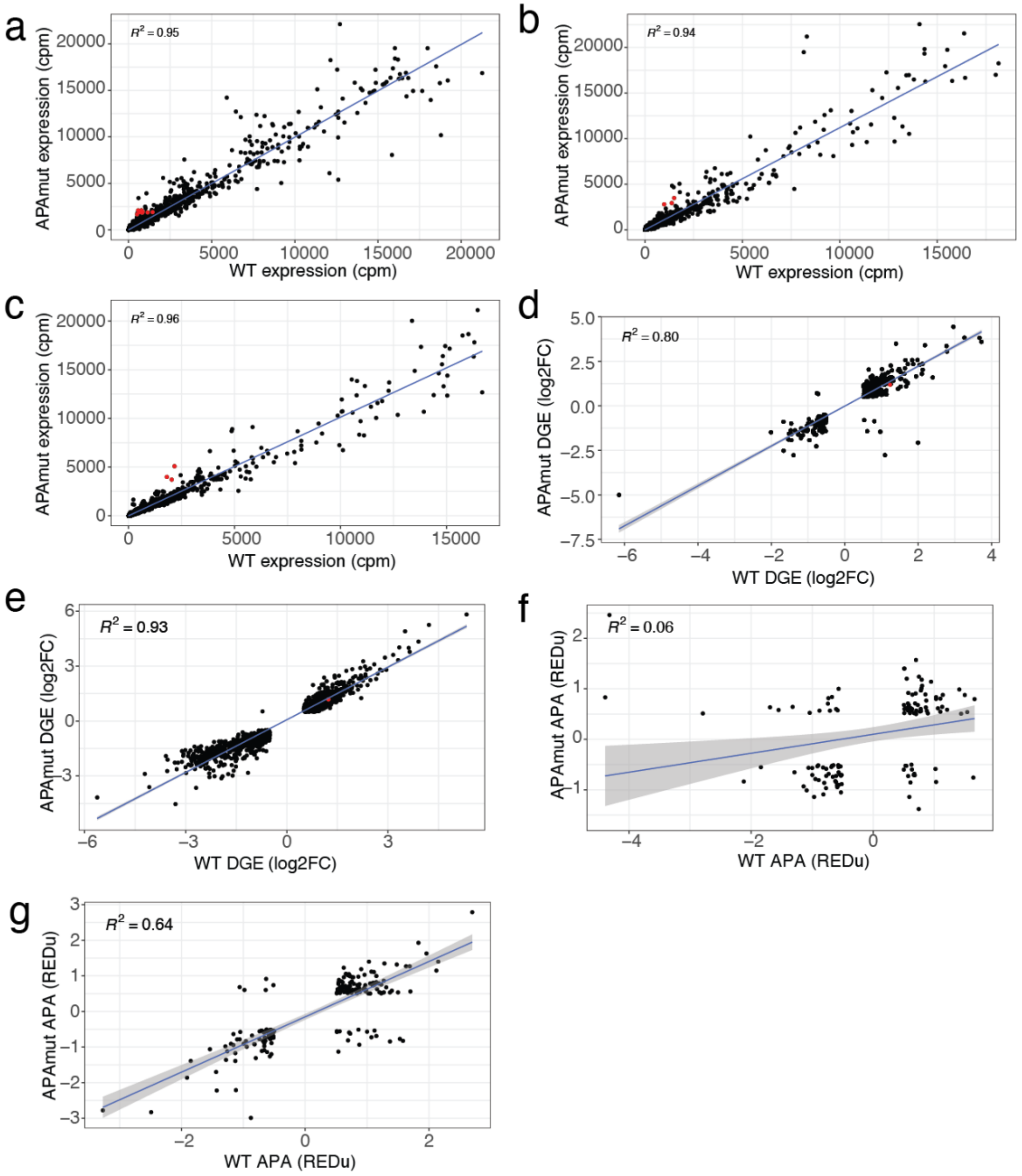

Supplemental Figure 3. Wild-type and APA-mut HepG2 cells have similar RNA expression and processing. Gene expression is highly correlated in (a) untreated lines, (b) 4 hours post-IL-6 exposure and (c) 24 hours post-IL-6 exposure. Genes identified as significantly differentially expressed (DGE) between untreated and (d) 4 hours IL-6 treatment or (e) 24 hours IL-6 treatment have correlated expression changes in wildtype and APA-mut cells. (f) At 4 hours post-IL-6 treatment alternative polyadenylation (APA) is not correlated between wild-type and APA-mut lines. (g) At 24 hours of IL-6 exposure, genes with differential polyadenylation is correlated between wild-type and APA-mut HepG2 cells.

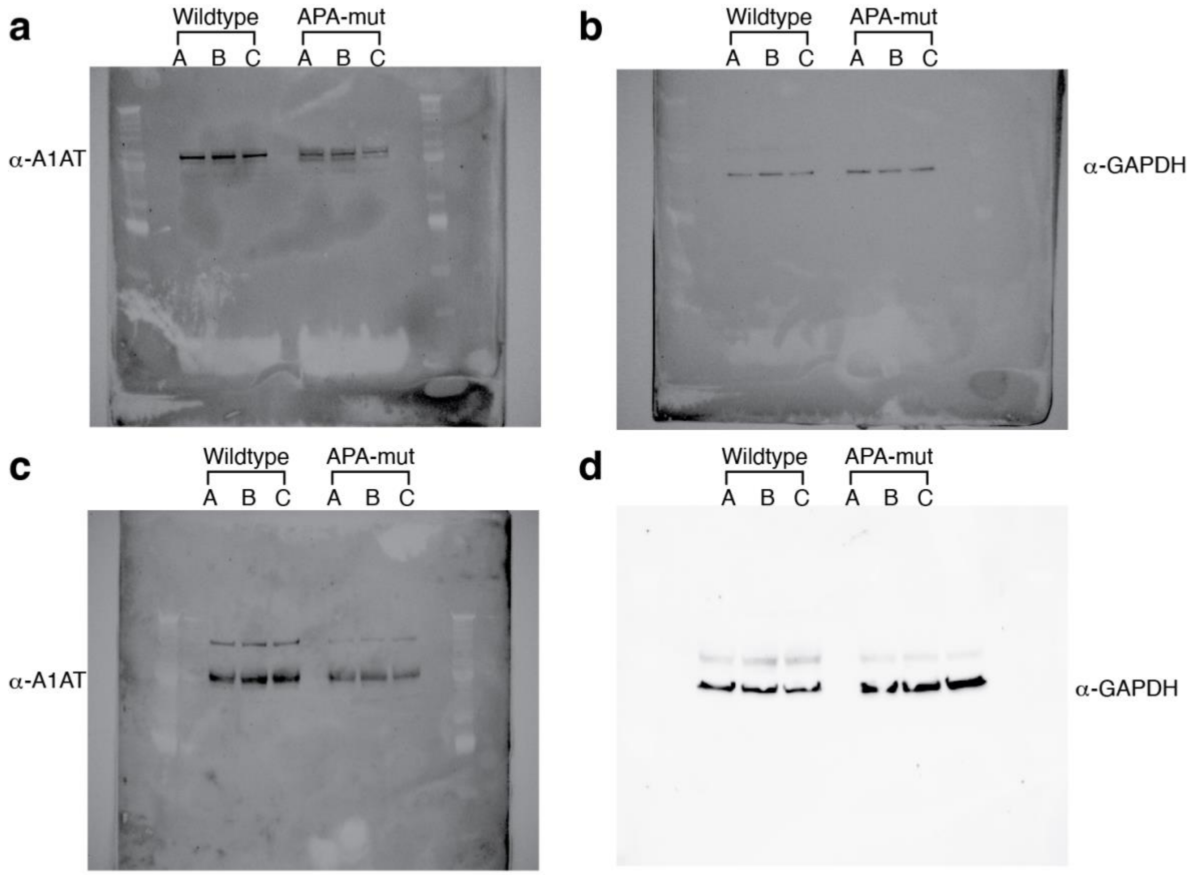

Supplemental Figure 4. Expression of endogenous A1AT from *SERPINA1* mRNA short and long 3'UTR isoforms. (a,c) A1AT protein levels are decreased in APA-mut CRISPR cells lines compared to wildtype HepG2 cells that predominantly express the long 3'UTR isoform. (b,d) GAPDH was used for normalization.

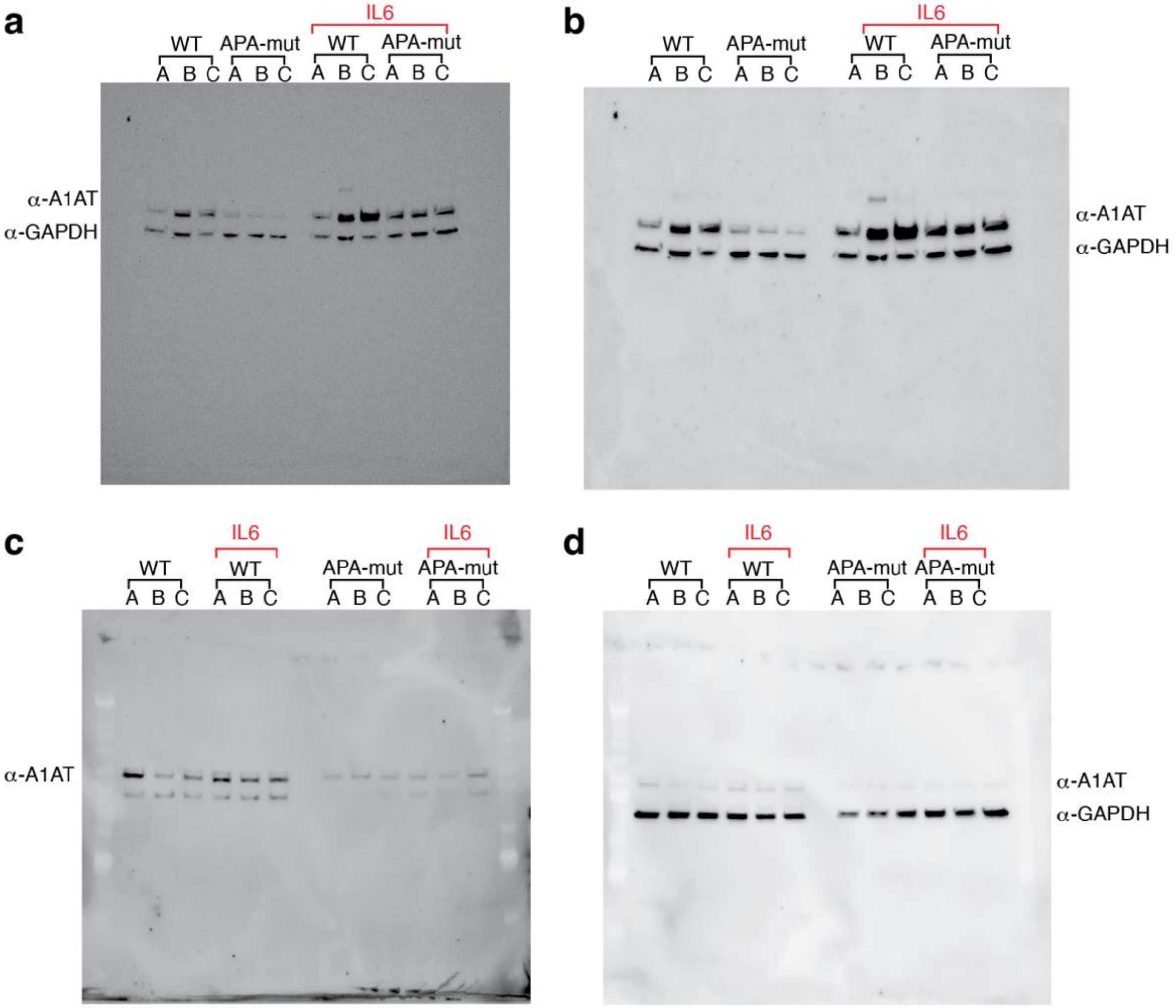

Supplemental Figure 5. Impact of IL6 treatment on endogenous A1AT protein. (a,c) IL-6 increases the amount of A1AT protein 24 hours after treatment. A1AT protein is decreased in APA-mut CRISPR cells lines in comparison to wildtype HepG2 cells. IL-6 treatment does not increase A1AT protein to the same extent in APA-mut cells. (b,d) GAPDH was used for normalization.

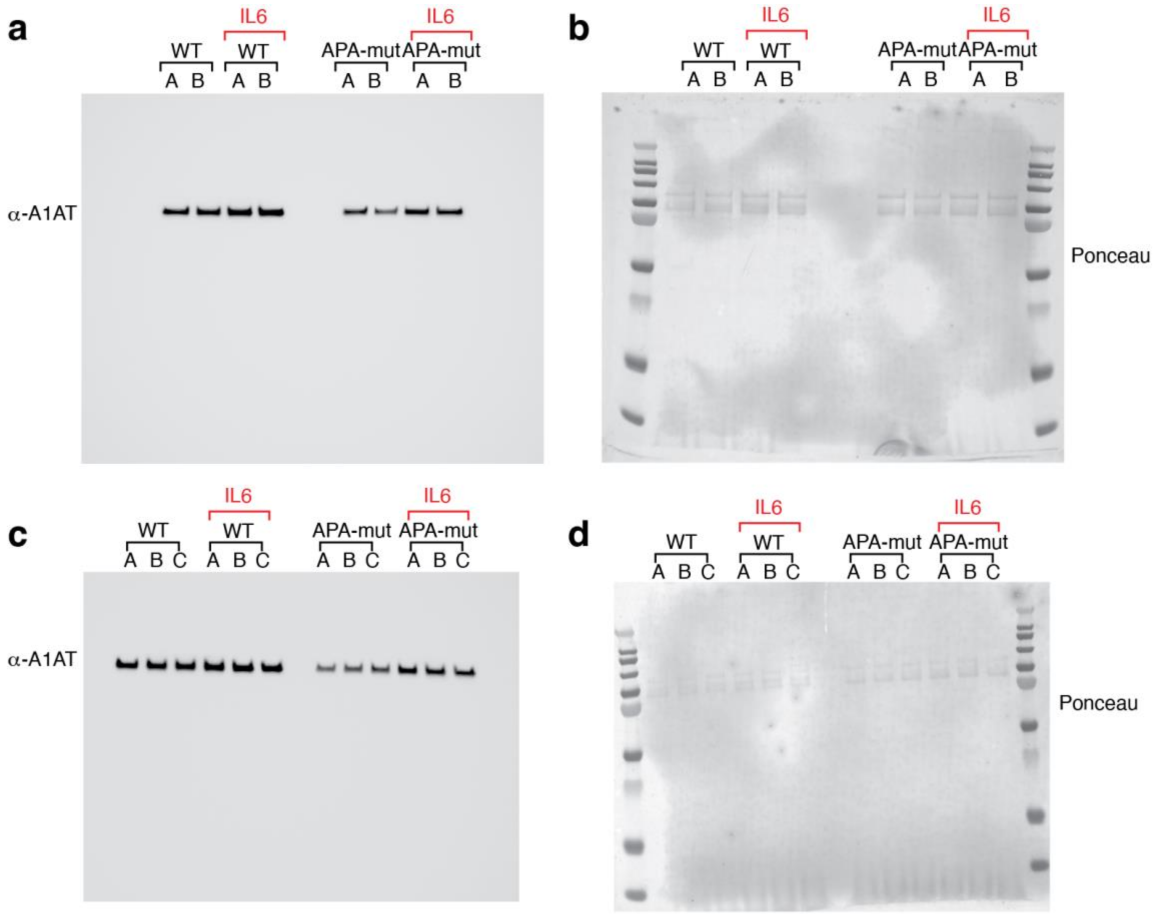

Supplemental Figure 6. Levels of secreted endogenous A1AT after IL-6 treatment in HepG2 and APA-mut cell lines. (a, c) A1AT protein levels in cell media are decreased in APA-mut CRISPR cells lines with and without IL-6 treatment. (b, d) Media was normalized by volume and Ponceau S staining was used to quantify loading.

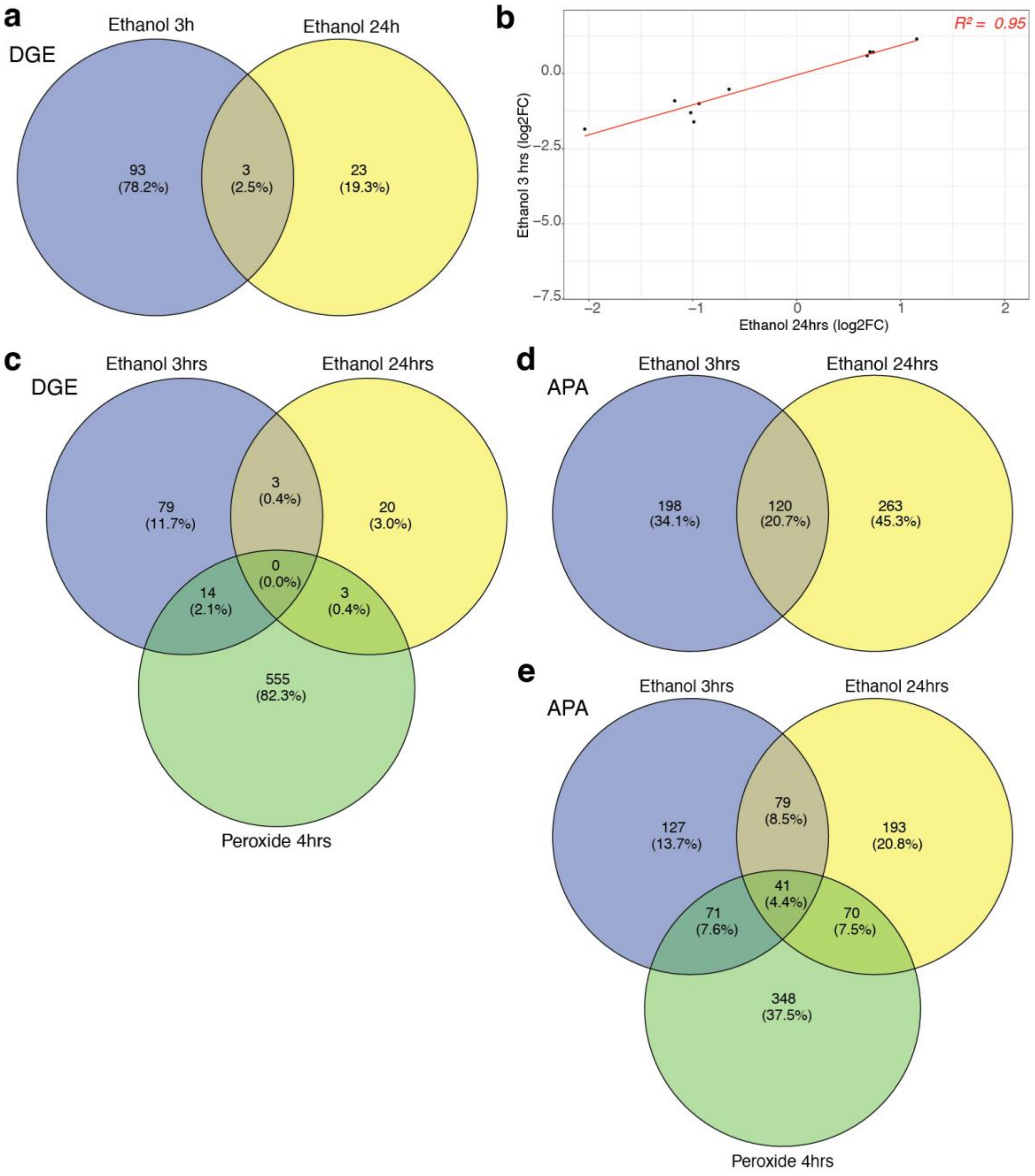

Supplemental Figure 7. Ethanol and peroxide change gene expression and polyadenylation. (a) Overlap of diffentially expressed genes between 3 and 24 hours of ethanol treatment is minimal but (b) expression is highly correlated for genes affected in both treatments (cutoff log2FC +/- 0.5). (c) More genes are affected by peroxide treatment than ethanol treatment and are not generally shared between treatments. (d) Overlap of significant alternatively polyadenylated genes between 3 and 24 hours of ethanol treatment. (e) Overlap of significant alternatively polyadenylated genes between peroxide treated cells and cells exposed to ethanol for 3 or 24 hours.

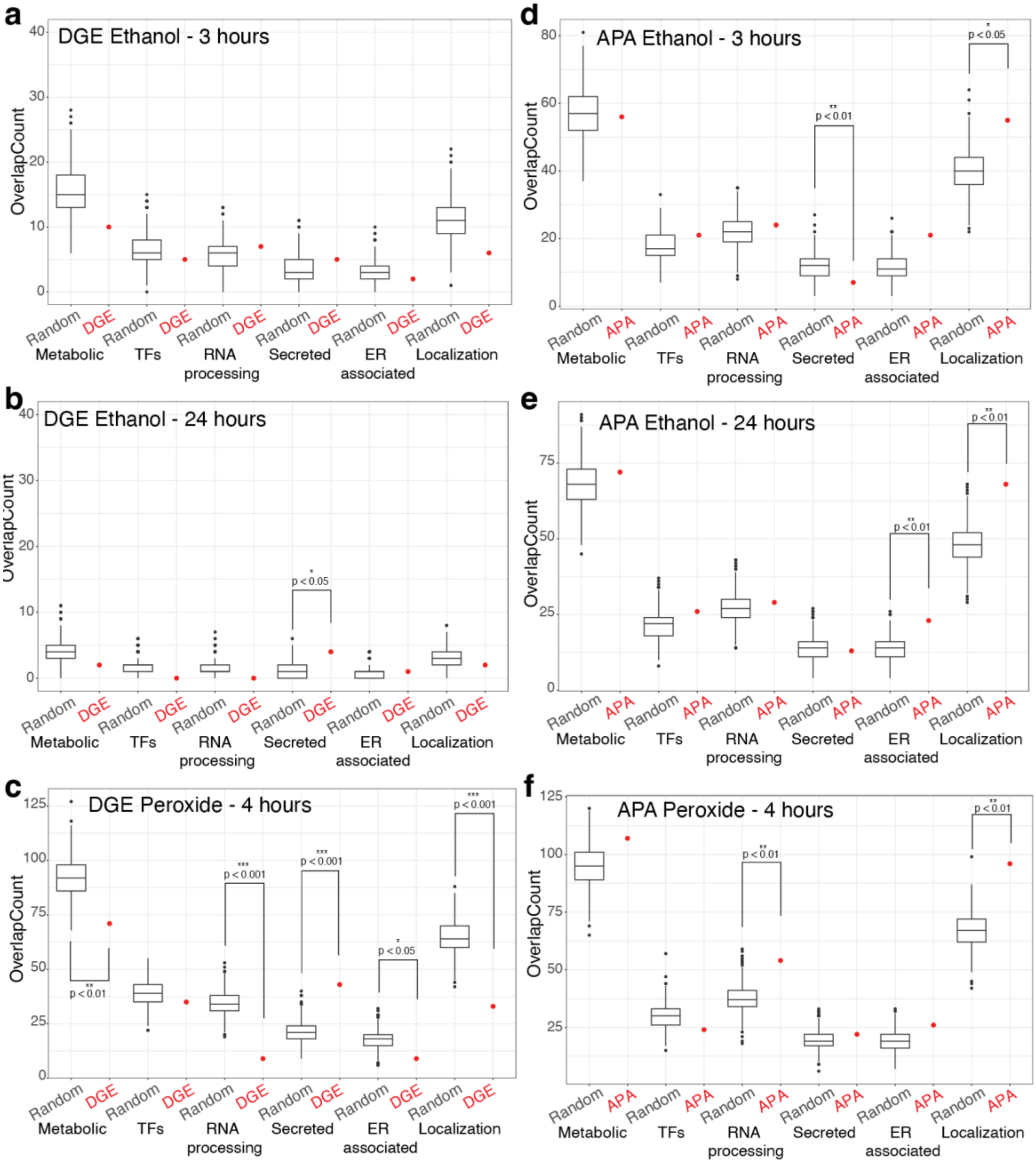

Supplemental Figure 8. Ethanol has a minor impact of gene expression and 3' processing compared to peroxide exposure. Select gene lists were tested for overlap between differentially expressed genes after (a-b) ethanol or (c) peroxide exposure compared to randomized expressed genes. The same lists were tested for overlap with alternatively polyadenylated genes after (d-e) ethanol or (f) peroxide exposure. Significance was calculated by permutation test and shown as $p<0.001$ (***), $p<0.01$(**) and $p< 0.05$ (*).

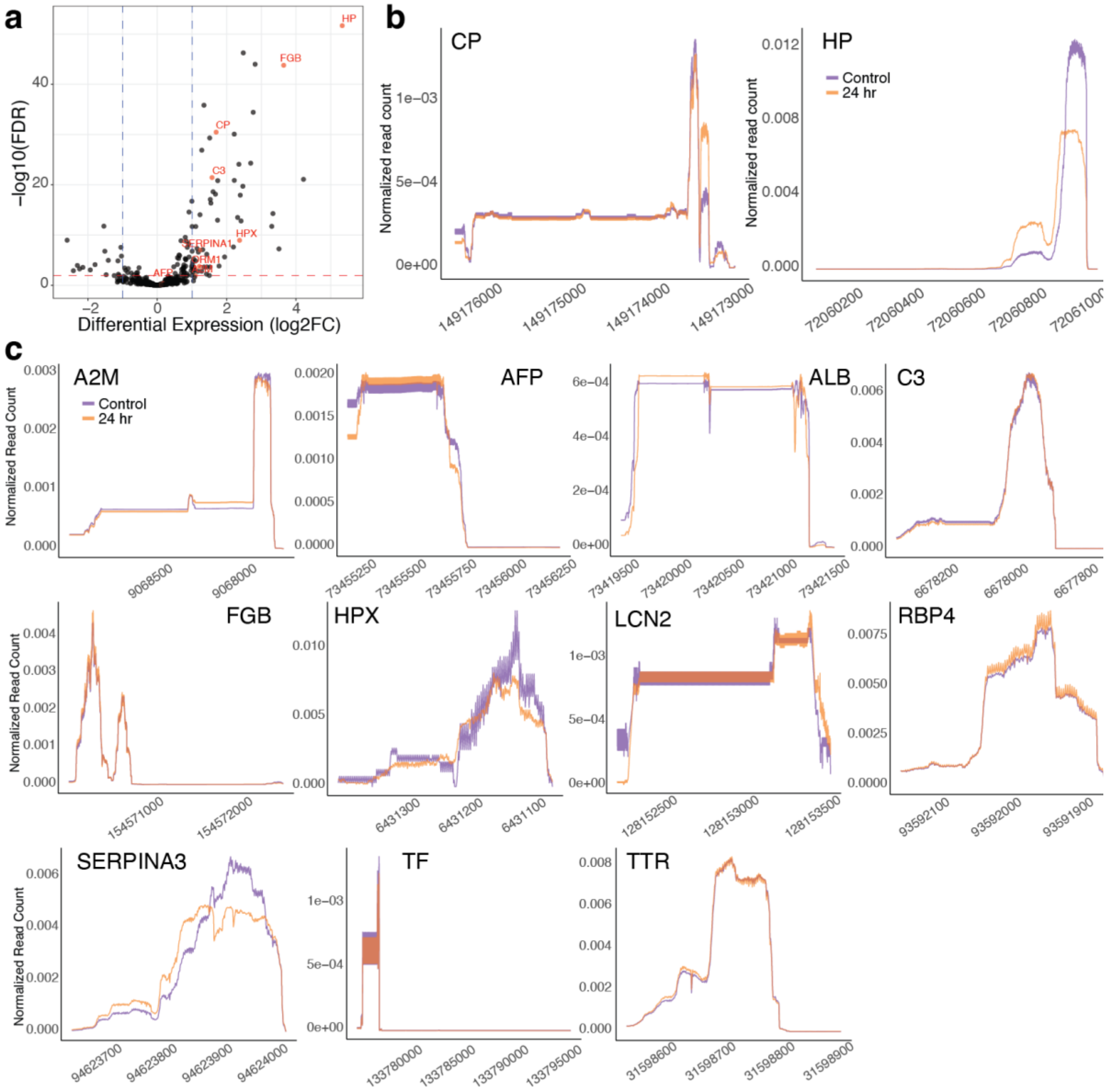

Supplemental Figure 9. Expression and polyadenylation of acute phase genes. (a) Most acute phase genes are upregulated 24 hours after IL-6 exposure (red) within the group of secreted genes. (b) Ceruloplasmin (CP) and haptoglobin (HP) show altered polyA site selection 24 hours after IL-6 exposure with individual quantification of region normalized read counts across the 3'UTRs. (c) The majority of expressed acute phase proteins do not have differential polyadenylation 24 hours after IL-[illegible]

Supplementary Table 1: Primers used for qRT-PCR.

	Sequence 5’ to 3’
ACTB_Fwd	CTGGAACGGTGAAGGTGACA
ACTB_Rev	AAGGGACTTCCTGTAACAATGCA
IL1R1_Fwd	ATGAAATTGATGTTCGTCCCTGT
IL1R1_Rev	ACCACGCAATAGTAATGTCCTG
FGG_Fwd	TTATTGTCCAACTACCTGTGGC
FGG_Rev	GACTTCAAAGTAGCAGCGTCTAT
GAPDH_Fwd	GGTGGTCTCCTCTGACTTCAACA
GAPDH_Rev	GTTGCTGTAGCCAAATTCGTTGC
BCL2_Fwd	AGGAAGTGAACATTTCGGTGAC
BCL2_Rev	GCTCAGTTCCAGGACCAGGC
GPX2_Fwd	GGTAGATTTCAATACGTTCCGGG
GPX2_Rev	TGACAGTTCTCCTGATGTCCAAA
